# Framing Subjective Emotion Reports as Dynamic Affective Decisions

**DOI:** 10.1007/s42761-023-00197-y

**Published:** 2023-07-10

**Authors:** Yi Yang Teoh, William A. Cunningham, Cendri A. Hutcherson

**Affiliations:** 1 Department of Psychology, University of Toronto, Toronto, ON Canada; 2 Department of Marketing, Rotman School of Management, University of Toronto, Toronto, ON Canada

**Keywords:** Subjective experience, Self-report, Computational models, Decision-making, Emotion, Affect

## Abstract

Self-reports remain affective science’s only direct measure of subjective affective experiences. Yet, little research has sought to understand the psychological process that transforms subjective experience into self-reports. Here, we propose that by framing these self-reports as dynamic affective decisions, affective scientists may leverage the computational tools of decision-making research, sequential sampling models specifically, to better disentangle affective experience from the noisy decision processes that constitute self-report. We further outline how such an approach could help affective scientists better probe the specific mechanisms that underlie important moderators of affective experience (e.g., contextual differences, individual differences, and emotion regulation) and discuss how adopting this decision-making framework could generate insight into affective processes more broadly and facilitate reciprocal collaborations between affective and decision scientists towards a more comprehensive and integrative psychological science.

## Affective Experiences

Subjective experience is fundamental to human affective processing (Adolphs, 2010; Barrett et al., 2007; Coan & Allen, 2007; Coppin & Sander, 2021; Cowen & Keltner, 2017; LeDoux & Hofmann, 2018; Quigley et al., 2014). Yet self-reports, the only direct measure of subjective affective experience, evoke both reverence and skepticism (Barrett & Westlin, 2021; LeDoux & Hofmann, 2018). Much affective science research relies on methodologies that assume self-reported emotion accurately indicates the presence of or changes in emotion states, permitting investigation of their (neuro)physiological and behavioral dynamics (Coan & Allen, 2007). Yet researchers have also characterized subjective reports as unreliable measures of affective dynamics that fail to cohere with physiological signatures of emotion (Barrett & Westlin, 2021; LeDoux & Hofmann, 2018; Quigley et al., 2014). Better specifying the psychological mechanisms that transform subjective emotion experiences into self-report ratings might help to resolve this tension. Yet surprisingly little work exists on this topic (Scherer & Moors, 2019).

In this review, we propose a two-part solution to this problem. First, consistent with a long history of theorizing in affective science, we view emotion reports as a class of affective decisions (Barrett, 2017; Berkovich & Meiran, 2022; Givon et al., 2020; Karmon-Presser et al., 2018) which take as evidence (among other possible sources) sensory signals (interoceptive: Critchley & Garfinkel, 2017; Terasawa et al., 2013; Wiens, 2005; and proprioceptive: Coles et al., 2019; Stepper & Strack, 1993) and situational appraisals (Lindquist & Barrett, 2008; Scherer & Moors, 2019; Singer-Landau & Meiran, 2021). Second, and more critically, following the example of others in the field (Givon et al., 2020; Karmon-Presser et al., 2018), we argue that viewing self-reports as evidence-based decisions allow us to draw on research from computational models of perceptual and value-based decision-making to resolve tensions over what affective self-reports mean, and reveal novel insights into the dynamics of affective experience. Specifically, we first establish that self-reports constitute a kind of decision. Next, we show how sequential sampling models of decision-making (SSMs: Forstmann et al., 2016) can disentangle subjective emotion experience from noisy and variable self-reports and identify distinct mechanisms through which moderators of subjective experience (e.g., individual differences, culture, and regulation) change self-reports. Finally, we argue that adopting this computational framework will allow for greater collaborative efforts between decision and affective scientists and theoretical synthesis between these fields.

## Self-Reports of Emotions as Affective Decisions

Conventional measures of self-reported affective experience often use (pseudo-)continuous numerical scales in combination with verbal labels to anchor interpretations of specific values (Coan & Allen, 2007; Jebb et al., 2021). Researchers use these scales to elicit and measure structured reflection on current, past, or future affective experiences by mapping them onto predefined choice options. Within a decision-making framework, one can conceive of these ratings as choices where participants evaluate which rating option best represents their affective states by constructing a task “value” for each option and comparing across them (Busemeyer et al., 2019; Givon et al., 2020; Karmon-Presser et al., 2018; Rangel et al., 2008).

For example, when you unexpectedly receive a gift from a close friend, you might feel both surprised and happy. While these categorical labels might not define the affective experience per se, they are useful ways to apprehend our subjective experiences and communicate them to others. If asked how you felt, the option to report these feelings as surprise might have a moderately high task-value because it captures your subjective feelings about the unexpectedness of the gift. Similarly, the option to report these feelings as happiness might also have a moderately high task-value because it captures your subjective feelings of being cared for by your friend. In contrast, the option to report these feelings as anger (if present) would have an extremely low task-value because it fails to capture any component of your subjective feelings. Thus, if presented with all three emotion labels as possible options (e.g., Do you feel happy, surprised, or angry?), you would likely self-report feeling surprised or happy but not angry. If presented only a choice between happy and angry, you would select happy.

Such a framework emphasizes how simply eliciting self-reported emotion fundamentally structures the interpretation of subjective affective experience as evidence for self-report. Here, we discuss how two specific structural features that vary across affective self-report measures (detection/differentiation vs. affect/emotion) might reconfigure this evidence construction process through which our affective states shape recorded self-reports.

First, self-reports may be structured to elicit (1) affective detection (whether a person experienced a specific affective state like anger) or (2) affective differentiation (which of a number of affective states, like anger or fear, a person experienced). Different specifications of the question can change what evidence is relevant for self-report (Kirkland & Cunningham, 2012). The former instructs participants to construct evidence for the presence/absence of an affective state; the latter instructs participants to accumulate evidence that discriminates between two possible affective states. For example, while interoceptive evidence about heart rate might drive self-reports of anger in presence/absence judgments, it is unlikely to be integrated as evidence for anger vs. fear because it might support the presence of both.

Second, self-reports are often used to measure both (1) more general dimensions of affective states (e.g., valence and arousal: Bradley & Lang, 1994; Kuppens et al., 2013; Russell, 1980) and (2) more specific and complex emotions (Watson et al., 1988). These distinct specifications could likewise shape the construction of evidence during ratings. While decisions about the unpleasantness of an experience (e.g., Was this experience unpleasant or not?) may rely more heavily on sensory evidence from interoceptive sources and less on specific situational appraisals, decisions about whether an experience constituted disgust (e.g., Did you feel disgusted (or not)?) might require careful consideration of both the interoceptive evidence and a much broader set of situational appraisals (Roseman et al., 1990).

However, common across these choice specifications is that the evidence construction process is psychologically and biophysically (e.g., neurally) constrained and thus noisy, due to random variation in the processing environment (both external and internal to the human body: Hilbert, 2012; Ratcliff, 2001). Consequently, recorded self-reports ultimately reflect a composite of both the underlying affective experience and variations in the decision-process (e.g., response biases, random noise).

## Sequential Sampling Models of Noisy Affective Self-Reports

Sequential sampling models formalize this decision-making framework by postulating that affective self-reports result from the weighted integration of noisy, experience-relevant evidence for each reporting option (Busemeyer et al., 2019; Forstmann et al., 2016; Givon et al., 2020). To deal with noise, these models assume individuals accumulate variable samples of their experience as evidence over time towards thresholds for various response options, with the ultimate choice and time taken to make that choice resulting from which threshold the accumulated evidence crosses first, and when. Thus, to deploy these models, measurements of self-reports should include not only the content of the self-report but also the time it takes participants to make those reports to capture the dynamics of the evidence accumulation process.

Inspired by others in the field (Givon et al., 2020; Karmon-Presser & Meiran, 2019), we likewise propose here that experiential evidence is driven by the weighted sum of inputs from a number of sources, including but not limited to interoception, proprioception, appraisals, and action tendencies (Eq. 1), which in turn drives subjective reports: more intense experiences generate stronger evidence for one reporting option over others, which subsequently leads to faster, more consistent reporting of that option. For example, when asked to report whether or not we felt anger (detection), we might recognize as anger an experience where we felt our heart racing (interoceptive evidence), our jaw clenching (proprioceptive evidence), an urge to punch something (action evidence), and wronged by another person (appraisals), weighing them all similarly. Importantly, these weights not only identify whether a source of information is relevant but also how relevant for self-reported subjective experience. As discussed above, each of these sources of evidence may be more or less informative for deciding between self-report options depending on the structure of the question eliciting the self-report.

1 $$\begin{array}{c}{evidence}_{i}={w}_{interoception:i}\left({x}_{interoception}\right)+{w}_{proprioception:i}\left({x}_{proprioception}\right)+\\ {w}_{appraisal:i}\left({x}_{appraisal}\right)+{w}_{action:i}\left({x}_{action}\right), i\in \left\{response options\right\}\end{array}$$

Differences in the context of self-report could also shape the relative contribution of random noise to the resulting decisions by changing the overall evidence-threshold for response. Increasing the thresholds makes self-reports more resistant to noise but result in longer decision times. Reducing thresholds makes reports faster but more subject to random noise (Bogacz et al., 2010). Additionally, asymmetrical thresholds for self-report options also systematically bias responses independent of the underlying affective experience (Forstmann et al., 2010; Leite & Ratcliff, 2011). Response options with lower thresholds are chosen more quickly and frequently because less evidence is required to cross the threshold but are more likely to be chosen in error (i.e., in contradiction to the overall evidence) due to noise.

**Fig. 1:**
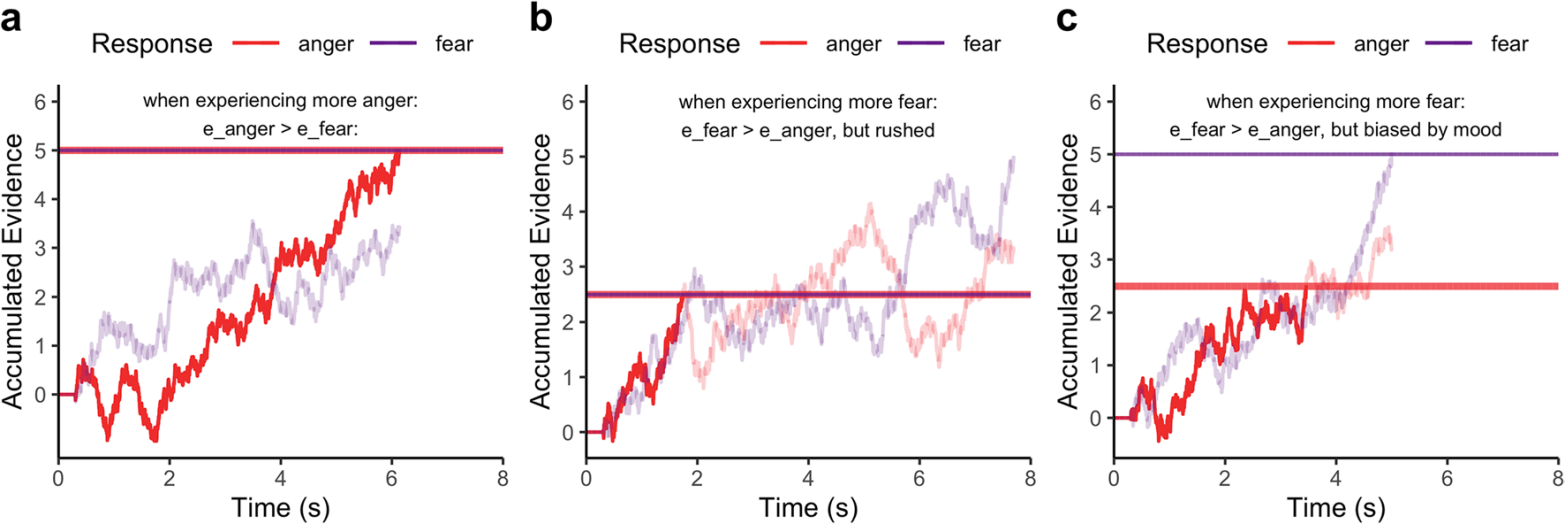
Simulations from a sequential sampling model of a binary choice categorizing an experience as anger or fear. Each plot depicts a simulated race between two accumulators using a racing diffusion model (Tillman et al., 2020), with two static horizontal lines representing the evidence thresholds for each choice option (i.e., anger or fear) and two variable solid lines representing the respective evidence accumulated over time. Simulations depict an example of **a** when a person categorizes their emotion experience as anger due to stronger evidence for the anger option (e_anger > e_fear), **b** when a person categorizes their emotion experience as anger despite stronger evidence for the fear option (e_fear > e_anger) because they expedited responses and lowered the threshold for choices, and **c** when a person categorizes their emotion experience as anger despite stronger evidence for the fear option (e_fear > e_anger) because their prior mood selectively lowered the threshold of evidence for the anger option

Take for example someone’s decision to self-report their negative experience with an aggressive stranger as anger rather than fear (see Fig. 1). One explanation for this report might be appraisals that the stranger’s aggression is unjustified, providing evidence for anger rather than fear (Kuppens et al., 2003; Lindquist & Barrett, 2008). Another possibility is that they actually appraised the stranger as a threat and thus indicative of fear but responded in error due to a combination of random noise and lack of response caution. A third possibility is that they were already in an irritated mood at the time of response (Schmid & Schmid Mast, 2010), priming their responses towards anger by selectively lowering the threshold of evidence for the anger option. Sequential sampling models can quantify the degree to which each of these mechanisms drive responses to explain and predict variability and stability of self-reports across contexts.

Critically, while we exclusively used examples with only binary self-report options for simplicity, sequential sampling models can also capture decisions between multiple options and even continuous scales (Brown & Heathcote, 2008; Evans et al., 2019; Heathcote et al., 2022; Kvam, 2019; Moran et al., 2015; Ratcliff, 2018; Ratcliff & Rouder, 1998; Roberts et al., 2023; Tillman et al., 2020). This means that principles of our decision-making framework may be applied equivalently across a variety of self-report measures, so long as response times are measured simultaneously.

## Applications of a Sequential Sampling Framework

In practice, application of these models requires multiple instances of self-report to varied affective stimuli. Researchers interested in determinants of affective experience could generate this variability by presenting a range of affective stimuli that differ on a few specific and quantifiable dimensions (e.g., physical color, psychological threat) and recording the content and timing of self-reports. Sequential sampling models fit to these data could then identify how these dimensions shape the affective dynamics specific to an individuals’ self-reports. By aggregating the model’s parameters across a sample of participants, researchers could then make inferences about (in)consistencies between individuals (Wiecki et al., 2013).

While the full potential of a sequential sampling framework remains to be tested, recent studies have begun to explore its utility in characterizing subjective affective experiences (Berkovich & Meiran, 2022; Givon et al., 2020). In these studies, participants indicated whether emotionally evocative images made them feel pleasant or not, and the authors modeled their choices and response times using sequential sampling models. Model-fitting in these studies revealed unexpected asymmetries in the way people experience negative and positive affect: people’s self-reports were not only more sensitive to negative experiences compared to positive experiences (Givon et al., 2020), but also more certain about the intensity of these negative experiences (Berkovich & Meiran, 2022). In other words, people accumulated evidence more quickly and with less noise for negative compared to positive experiences. Consequently, while evidence for negative experiences scaled linearly with intensity, for a positive experience to generate an evidence signal twice as strong as another positive experience (relative to the noise), it needed to be more than twice as intense. These findings suggest that people generally form stronger and more precise impressions of negative experiences, possibly explaining why they sometimes learn faster from negative compared to positive feedback (Gershman, 2015).

Moreover, these models hold great promise for affective scientists because they identify multiple target processes through which individual differences, context, and regulatory strategies can shape self-reports of affective experience. In a recent paper, Givon et al. (2023) applied these models to elucidate the precise mechanism that underlies previously reported gender differences in affective experience. By distinguishing between the evidence and thresholds for self-reports of valence, they found that women generated significantly stronger evidence towards negative stimuli compared to men but had similar thresholds for responding. These findings suggest that women were not a priori biased towards reporting all stimuli as negative, but rather may actually experience negative stimuli more intensely than men. These results raise important questions about the source of these gender differences in evidence construction. Do women have stronger physiological responses to negative stimuli than men or simply weight equivalent responses more heavily? Alternatively or in addition, do women recruit a different set of appraisals as evidence for these self-reports? Answers to these questions may help us better understand consequential gender differences in the reported prevalence of affective disorders (Altemus et al., 2014), since coherence between physiology and self-reported affective experiences have been found to predict subjective well-being (Brown et al., 2020).

Similarly, these models could better characterize contextual differences in self-reported emotional experience, like those between cultures, and whether they derive directly from differences in the appraisals involved in the evidence generation process (Imada & Ellsworth, 2011; Roseman et al., 1995; Scherer, 1997), or thresholds of evidence for specific response options due to cultural norms (Matsumoto, 1990; Mesquita & Walker, 2003). Alternatively, these models could also advance research on how distinct emotion regulation strategies shape subjective experience and self-reports (McRae et al., 2012; Troy et al., 2018). For example, experimental demand when instructing people to regulate could decrease reports of emotion not because internal experience changes, but because it increases the threshold of evidence required for reporting any emotion (positive or negative), or biases people to respond positively. A sequential sampling framework allows researchers to better explore how various emotion regulation strategies target different substrates of the affective process during self-reports of experience (Gross, 2015). This, in turn, would not only better characterize the specificity and efficacy of emotion regulation strategies but also open up investigations into more complex regulatory strategies that target multiple affective processes or combine multiple techniques (Ford et al., 2019).

## Future Directions

At the same time, the quality of inferences about the dynamics of affective self-report will also improve as the sophistication of computational models improves. Newer sequential sampling models enable researchers to segment the evidence accumulation process into distinct stages that weight evidence in different ways at different times (Diederich & Trueblood, 2018; Maier et al., 2020), and incorporate richer forms of data, like eye-movements, to identify on a moment-by-moment basis what evidence is being prioritized by visual attention (Krajbich et al., 2010; Teoh et al., 2020). Such approaches could be easily adopted by affective scientists who seek to understand how moment-to-moment changes in affective experience drive self-reports, by grounding evidence construction in temporally precise measures of physiological activity like skin-conductance response or cardiac inter-beat interval (Butler, 2017). Future models could further be developed using multi-modal methods combining physiological recording and eye-tracking to understand how interoceptive processes and visual attention jointly drive the subjective experience and self-report of emotions.

Additionally, while we have only discussed the utility of a decision-making framework in uncovering the temporal dynamics that lead up to a single self-report thus far, our approach also offers insight into how affective experiences may evolve from one self-report to the next. Recent research suggests that naming affective experiences impedes subsequent attempts to regulate these experiences (Nook et al., 2021). By combining this observation with decision-making research on confirmation biases (Chaxel et al., 2013; Navajas et al., 2016; Talluri et al., 2018), sequential sampling models provide a means to test the idea that emotion-naming selectively constrains patterns of attention and appraisals which leads to emotional rigidity during subsequent self-reports (Moran et al., 2015; Turner et al., 2021), formalizing theories about the iterative nature of affective processes (Cunningham et al., 2013; Ford et al., 2019; Gross, 2015; Gross & Barrett, 2011).

In light of accelerating interest in the role of affective states on cognition and behavior across broad swaths of psychological science (Dukes et al., 2021; FeldmanHall & Heffner, 2022; Lerner et al., 2015; Phelps et al., 2014; Roberts & Hutcherson, 2019), we hope that our paper here highlights the utility of a reciprocal approach to understanding subjective affective experience, and affective processes more generally, by drawing on insights from decision-making research. We believe that these kinds of collaborations between affective and decision scientists will spur continued discoveries in the respective fields and contribute to a more comprehensive and integrative psychological science.
